# Effect of Travel Restrictions of Wuhan City Against COVID-19: A Modified SEIR Model Analysis

**DOI:** 10.1017/dmp.2021.5

**Published:** 2021-01-08

**Authors:** Yue Li, Shike Hou, Yongzhong Zhang, Junfeng Liu, Haojun Fan, Chunxia Cao

**Affiliations:** 1 Institute of Disaster Medicine, Tianjin University, Tianjin, P.R. China; 2 Department of Mathematics, Renai College, Tianjin University, Tianjin, P.R. China

**Keywords:** COVID-19, SEIR model, export risk, migration indexes

## Abstract

**Objective:**

Since December 2019, a new coronavirus viral was initially detected in Wuhan, China. Population migration increases the risk of epidemic transmission. Here, the objective of study is to estimate the output risk quantitatively and evaluate the effectiveness of travel restrictions of Wuhan city.

**Methods:**

We proposed a modified susceptible-exposed-infectious-recovered (SEIR) dynamics model to predict the number of coronavirus disease 2019 (COVID-19) symptomatic and asymptomatic infections in Wuhan. And, subsequently, we estimated the export risk of COVID-19 epidemic from Wuhan to other provinces in China. Finally, we estimated the effectiveness of travel restrictions of Wuhan city quantitatively by the export risk on the assumption that the measure was postponed.

**Results:**

The export risks of COVID-19 varied from Wuhan to other provinces of China. The peak of export risk was January 21-23, 2020. With the travel restrictions of Wuhan delayed by 3, 5, and 7 d, the export risk indexes will increase by 38.50%, 55.89%, and 65.63%, respectively.

**Conclusions:**

The results indicate that the travel restrictions of Wuhan reduced the export risk and delayed the overall epidemic progression of the COVID-19 epidemic in China. The travel restrictions of Wuhan city may provide a reference for the control of the COVID-19 epidemic all over the world.

A novel coronavirus was identified in December 2019 in Wuhan city of Hubei Province, China. Chinese authorities identified that the causative agent was a new coronavirus, and later, the disease was officially named by the World Health Organization (WHO) as coronavirus disease 2019 (COVID-19), which received considerable global attention.^1,2^ On January 30, 2020, WHO announced that it would list the new coronary pneumonia epidemic as an “emergency public health event of international concern”.^3^ As of June 23, 2020, a total of 85,098 confirmed and 4647 deaths had been reported in China.^4^ And a total of 9,081,678 people are infected worldwide.^5^

Epidemiological evidence shows that COVID-19 has a strong infectious ability.^6,7^ The mild patients infected by the novel coronavirus are the main source of infection; asymptomatic patients can also be infectious sources, and they are not easy to find.^8,9^ These characteristics bring challenges for the prevention and control of the epidemic.

In the early stage of the epidemic in China, most of the confirmed cases in areas other than Wuhan had a history of travel to or residence in Wuhan and its surrounding areas within 14 d before the onset of the disease.^9,10^ The total number of cases reported from each province in January 2020 was strongly associated with the total number of travelers from Wuhan.^11^ These studies suggested that population migration from Wuhan increases the risk of epidemic transmission to other places in China.^12^ Further spread of COVID-19 was of great concern in view of the upcoming Spring Festival 2020 (January 25), during which there are typically 3 billion travel movements over the holiday period.^13^ Output risk assessment from Wuhan to other places in China is beneficial to analyze and control the COVID-19 epidemic.

To stop transmission of the virus, the Chinese government implemented a series of intervention measures. On January 20, 2020, the management of COVID-19 was upgraded to the highest level pertaining to class A infectious diseases. On January 21, 2020, the Health Commission officially started to release daily disease information on the website. To control the epidemic in Wuhan and delay the spread of the epidemic progression to other regions, Wuhan New Coronavirus Infection Pneumonia Epidemic Prevention and Control Headquarters issued Circular No. 1 that from 10:00 on January 23, 2020, public transportation in Wuhan was temporarily suspended, and the passage away from Wuhan was temporarily closed.^14^ Other public health interventions were also implemented. However, in the early days of the outbreak, the effectiveness of these interventions was questionable, especially the travel restrictions of Wuhan city.

As recognized by the WHO, mathematical models have played a key role in outbreak forecast and informing evidence-based decisions by health policy-makers.^15^ A large number of infectious disease dynamics models have so far been publicly released for this epidemic, which can be used for prediction of epidemic trend, development of policy, and evaluation of measures.^16,17^ However, few models have been applied to estimate the risk of symptomatic and asymptomatic infections to evaluate the effectiveness of the travel restrictions of Wuhan city in early epidemic of China. Hu et al. estimated the risk of Wuhan output based on the number of reported cases and the emigration index without model study.^18^ The previous studies assessed the risk of Wuhan output without considering the infectivity of asymptomatic patients.^19,20^

To provide scientific basis for prevention and control in the initial stage of COVID-19, the present study estimated the output risk quantitatively and evaluated the effectiveness of travel restrictions of Wuhan city based on the modified susceptible-exposed-infectious-recovered (SEIR) infectious disease dynamic model and migration data.

## Methods

### Data

Data were extracted from the following sources: official websites of the National Health Commission of the People’s Republic of China and provincial Health Commissions, WHO Coronavirus Disease Dashboard, and published literature. Data information included the reported cumulative number of laboratory-confirmed cases, deaths, and discharges. The confirmed cases were diagnosed according to *the Diagnostic and Treatment Guidelines for COVID-19 (Version 3-4)* issued by the National Health Commission of the People’s Republic of China.^21,22^ A case is a confirmed case if the patient has 1 of the following etiological findings: a positive result of COVID-19 nucleic acid test using real-time fluorescent reverse transcriptase polymerase chain reaction (RT-PCR) a genetic sequencing result highly homologous to the known COVID-19 virus. Asymptomatic infections are defined as those with no clinical symptoms whose specimens test positive for the pathogen of COVID-19 or serum specific immunoglobulin (Ig) M antibody.

In addition, we obtained daily indexes and the proportion of emigration from Wuhan to 30 provinces in China (excluding Hubei, Taiwan, Hong Kong, and Macao, China) released by Baidu Migration (https://qianxi.baidu.com/?from=shoubai, from December 31, 2019 to January 30, 2020).

### The Mathematical Model and Parameter Estimation

**Figure 1.**
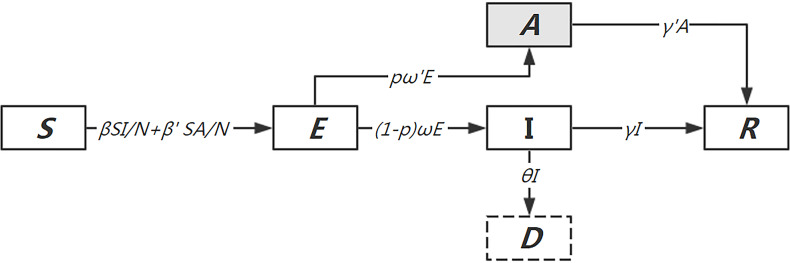
Diagram of the model adopted for COVID-19 simulation based on the epidemiological characteristics.

Based on the epidemiological characteristics of COVID-19, we proposed a modified SEIR dynamics model to simulate the transmission in Wuhan in 40 d from December 31, 2019. We stratified the populations as susceptible (*S*), exposed (*E*), asymptomatic infections (*A*), infected person with symptoms (*I*), recovered (*R*), and deceased (*D*), and further considered asymptomatic infections as the source of infection (Figure 1).

The transmission dynamics model describing the status of each compartment is shown in the following differential equations.
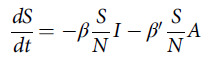


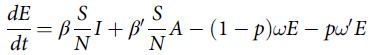


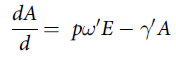


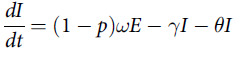


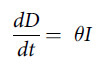


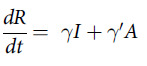



We surveyed the available literature to identify likely ranges of parameters of COVID-19 (Table 1). On December 31, 2019, there were 27 reported COVID-19 cases with symptoms in Wuhan. The model used this day as the 0^th^ day, and set 27 cases as the number of I on the 0^th^ day. The *β* was derived from the basic infection number (*R*_*0*_), and the calculation formula of β was:
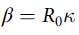



**Table 1.** Parameter definition and estimation of modified SEIR dynamic model in COVID-19 epidemic of Wuhan in China

Parameter	Definitions	Estimated Value	Range of value	Data Source
*β*	Probability of infection by symptomatic infections	0.45	0.19-0.65	7, 26–28
*β'*	Probability of infection by asymptomatic infections	0.30	2/3*β	9, 24
*p*	Proportion of asymptomatic infections	0.16	0.15-0.20	25
*ω*	Reciprocal of incubation period of symptomatic cases	0.17	0.14-0.33	9
*ω'*	Reciprocal of incubation period of asymptomatic cases	0.25	0.14-0.33	25
*γ*	Reciprocal of infectious period of symptomatic cases	0.05	0.05-0.09	29,30
*γ'*	Reciprocal of infectious period of asymptomatic cases	0.10	0.10-0.17	29
*θ*	Case fatality rate	0.06	–	Epidemic data

where the value range of *R*_*0*_ was 1.9-6.5 and the *κ* denoted the reciprocal of the infectious period of COVID-19, which was 1/10.^23^ We repeated the primary analysis across the range of *β* derived from the *R*_*0*_, from 0.19 to 0.65.

The previous epidemiological investigation suggested the incubation period is 1 to 14 d, mostly 3 to 7 d.^9^ The value of *θ* referred to the average case fatality rate in January in Wuhan. The value of *γ* referred to the infectious period of COVID-19 symptomatic cases. The estimation of relevant parameters (*p*, *β*, *ω'*, and *γ'* ) of COVID-19 asymptomatic cases were based on previous studies.^9,24,25,29,30^

We explored an optimal set of parameters in the ranges identified from the previous related studies by fitting the official daily reported cumulative number of COVID-19 cases from January 21, 2020 (the date of starting to release daily disease information) for the next 20 d. The root mean square error (RMSE) was adopted as the criterion of parameter setting, ie, fitting error, to measure the difference between the number of infected individuals generated by the model and the official daily reported cumulative number of COVID-19 cases in the early epidemic situation in Wuhan.
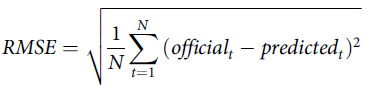



where the value of *official*_*t*_ was the daily reported cumulative number of COVID-19 cases. The value of *predicted*_*t*_ was the number of infected individuals generated by the model. N was 20.

### Calculation of Output Risk Coefficients

On the basis of the theoretical prediction values of *A* and *I* in the early stage of the COVID-19 epidemic in Wuhan and the emigration indexes from Wuhan to other provinces, we evaluated the output risk coefficients of each province from Wuhan. The formula for calculating the risk coefficients from Wuhan on *t* day of *i* province was established as follows.
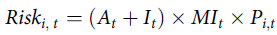



Where *Risk*_*i,t*_ was the output risk from Wuhan on the *t* day of the *i* province, *A*_*t*_ and *I*_*t*_ represented the number of asymptomatic and symptomatic patients in Wuhan city on the *t* day, respectively, *MI*_*t*_ denoted the emigration index of Wuhan city on the t day, and *P*_*i,t*_ was the percentage of migrants in Wuhan who moved to province *i*.

The formula for calculating the risk standardized coefficients from Wuhan on the *t* day of *i* province as follows:
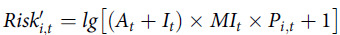



We calculated the total output risk coefficients for a period of time in *i* province, using the following formula:
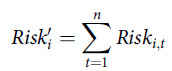



Spearman correlation analysis was conducted between the total risk factors of the epidemic situation and the reported cumulative numbers of confirmed cases in each province from January 1 to January 23.

### Estimated Effectiveness of Travel Restrictions of Wuhan City

Assuming that the travel restrictions of Wuhan city were postponed, we calculated the risk coefficients of the COVID-19 epidemic in each province. The Wuhan emigration indexes after the travel restrictions of Wuhan city in 2020 referred to the same period in 2019. Because the travel intensity during the Spring Festival was higher than daily, we selected the emigration index for the same period of the lunar calendar in 2019 as the basis for calculation.

### Data Analysis

MATLAB software (version R2019a) was used for building the modified SEIR model and calculating the epidemic risk coefficients of each province. We used SPSS software (version 26.0) to analyze the correlation between the reported cumulative numbers of cases and the total risk coefficients in each province. All statistical tests were 2-sided and *P* < 0.05 was considered as statistically significant.

## Results

### The Distribution of Confirmed Cases and Population Emigration Index of Wuhan

**Figure 2.**
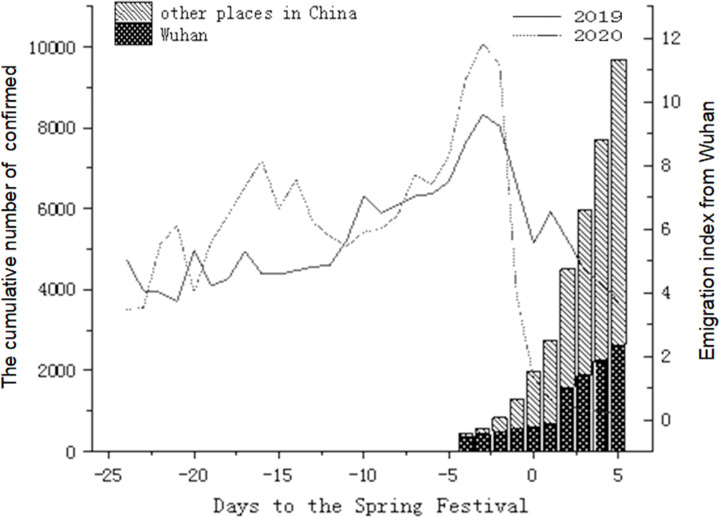
The distribution of confirmed cases and population emigration index of Wuhan (The population emigration indexes from Wuhan were in January 2020 and the same period of the lunar calendar in 2019. The Spring Festival was on January 25, 2020).

As of January 21, 2020, the number of confirmed cases reported in Wuhan accounted for 82.5% of the national total. The epidemic was spreading rapidly, with 2639 confirmed cases in Wuhan city, accounting for 27.23% of China as of 30 January. Before the travel restrictions of Wuhan city (January 23, 2020), the population emigration index of Wuhan showed an overall upward trend. After the closure of the city, the emigration index of Wuhan fell off a cliff (Figure 2).

### Forecast of the Initial Epidemic of COVID-19 in Wuhan Based on Modified SEIR Model

**Figure 3.**
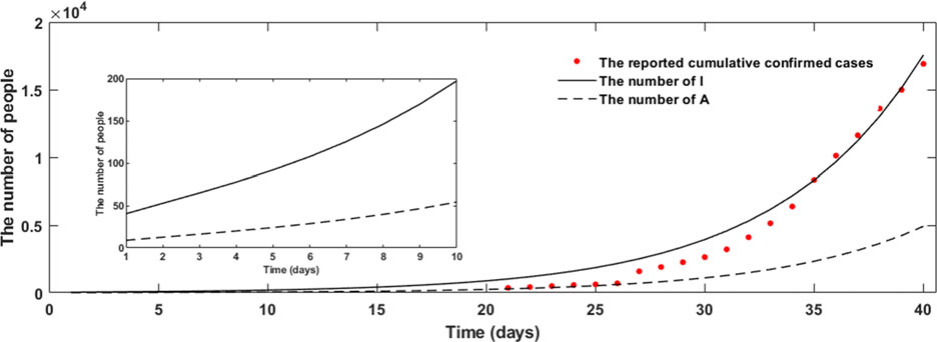
The theoretical predicted value of the number of symptomatic infection and asymptomatic patients based on the modified SEIR model (*I*: infected person with symptoms; *A*: asymptomatic infections).

The estimations showed that the cumulative numbers of *I* and *A* increased in January 2020 of Wuhan on modified SEIR model with the optimal parameters (Figure 3). The reported cumulative number of cases deviated from the theoretical prediction *I* value in January, 2020, which may be because many patients were missed by the surveillance system in the early stage of the COVID-19 epidemic. The simulation model showed the estimated numbers of symptomatic and asymptomatic infections were approximately 3939 and 1103, respectively, in Wuhan as of January 30, 2020.

### The Output Risk Assessment of COVID-19 From Wuhan to Provinces in China

**Figure 4.**
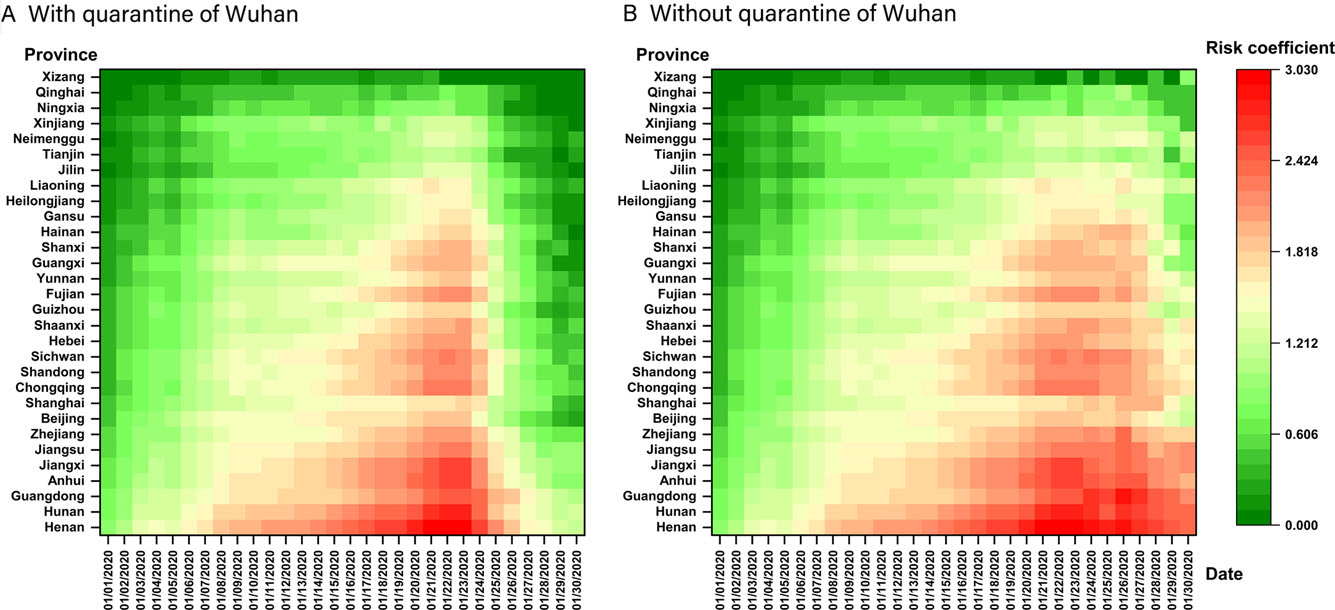
Standardized export risk of COVID-19 epidemic from Wuhan to provinces in China (The standardized risk coefficients of provinces ranged with travel restrictions of Wuhan city are presented in panel A in the early stage of the epidemic; The standardized risk coefficients of provinces ranged without travel restrictions of Wuhan city until January 30 are presented in panel B in the early stage of the epidemic.).

The standardized risk coefficients of each province ranged from 0 to 3.03 in the early stage of the epidemic (Figure 4A). The peak of epidemic export risk was from 21 to 23 January 2020 in Wuhan. The 3 provinces with the highest total risk coefficients were Henan, Hunan, and Anhui, and with the lowest were Xizang, Qinghai, and Ningxia from January 1 to 23, 2020.

**Figure 5.**
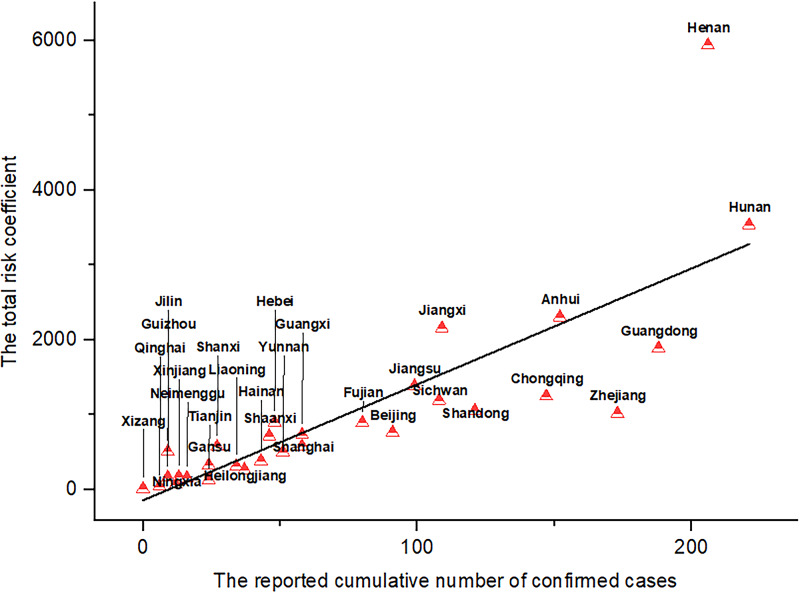
Spearman correlation coefficient between the standardized total input risk coefficients and the cumulative number of confirmed cases in provinces of China.

The correlation analysis of the total risk coefficients and the reported cumulative numbers of confirmed cases from January 23 to 30 showed that all correlation coefficients were statistically significant (*P* < 0.00). The total risk coefficients lagging by 5 days had the highest correlation with the reported cumulative numbers of reported cases in each province (Spearman correlation coefficient = 0.932) (Figure 5). We identified the provinces with a higher-than-expected level of infection, such as Zhejiang. On the other hand, there were some provinces with lower trends than expected (Henan).

### The Effectiveness of Travel Restrictions of Wuhan City Based on Output Risk Assessment

**Figure 6.**
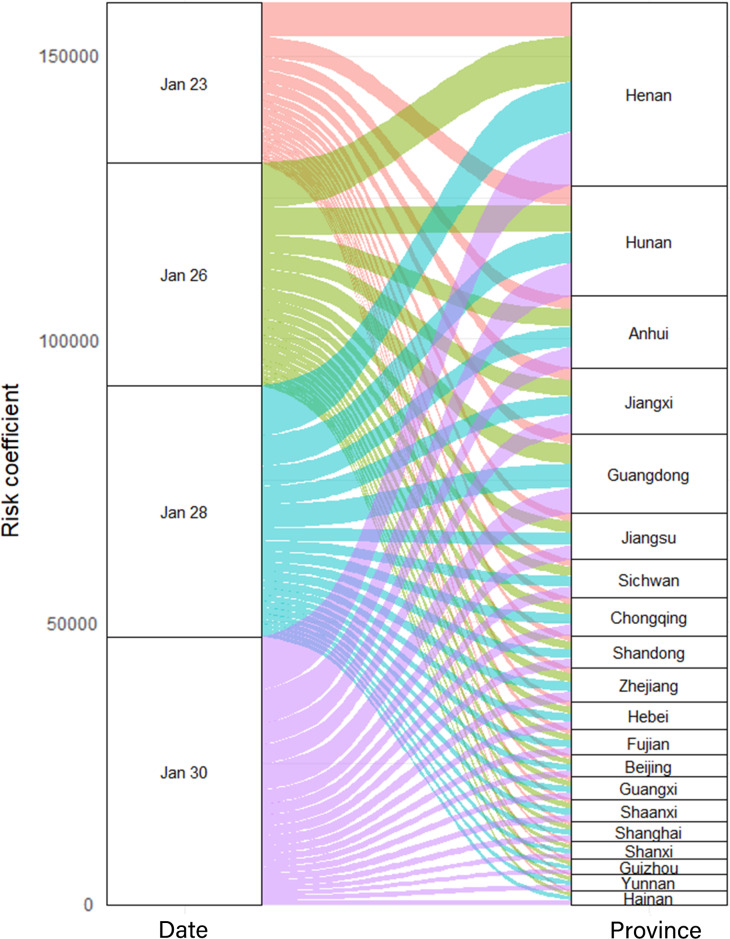
Export risk from Wuhan to part of the provinces (20 provinces with higher risk factors) when the travel restrictions of Wuhan city were implemented at different times.

Assuming that the travel restrictions of Wuhan city were not implemented until January 30, 2020, the other provinces would continue to be at the peak of COVID-19 output risk from January 23 to January 30 (Figures 4B). The risk factors of Henan, Hunan, and Guangdong province were the highest on January 30 (9445.25, 5900.16, and 4588.30) (Figure 6). Compared with the implementation of the travel restrictions of Wuhan city on January 23, the output risk factors would increase, respectively, 38.50%, 55.89%, and 65.63% assuming that this measure was postponed by 3 d (January 26), 5 d (January 28), and 7 d (January 30). The daily average increased risk coefficients were the highest from the January 26 to 28.

## Discussion

Evaluation and response of risk of crucial are of importance for infectious disease threats planning and control. In this study, we assessed the epidemic risk coefficients of each province based on the modified SEIR model and the Wuhan-to-province migration index provided by Baidu Migration. The response measure of output risk, travel restrictions of Wuhan city, was evaluated by the epidemic risk coefficients.

The epidemiological characteristics of infectious diseases are the basis for prevention and control of infection. The epidemiological characteristics of COVID-19 are partly different from severe acute respiratory syndrome (SARS) and Middle East respiratory syndrome (MERS). Asymptomatic infections are 1 of the infectious sources, accounting for 15.5%-20.2% of cases.^25^ The length of the infectious period and the ability of infection of asymptomatic patients need to be further studied.^9^ *The Pneumonia Prevention and Control Plan for New Coronavirus Infection (Third Edition)* issued by the National Health Commission of China stated that COVID-19 asymptomatic infections would be included in prevention and control management.^31^ On April 1, 2020, the Chinese authorities implemented a daily report system for asymptomatic patients to find these people to the greatest extent and reduce infection risk.^32^ We added asymptomatic infections and their output risks of patients and asymptomatic infections to increase the accuracy and application of the risk evaluation.

Forecasting and analyzing the spread of infectious diseases by dynamic model have demonstrated value in recent outbreaks by informing policy and epidemic management decisions in real-time outbreak response.^33^ Although many approaches to epidemic modeling exist, SEIR model was chosen for simplicity and speed of deployment.^34^ Given substantial uncertainty regarding input parameters for more complicated models and the need to communicate and iterate rapidly with decision makers, we opted not to incorporate social networks to model transmission dynamics. The results of this study showed that the theoretical predictions in January were quite different from the actual incidences due to uncertainty in diagnosis and other issues related to patient presentation at the beginning of the outbreak. There are some differences in the model parameters of the initial epidemic in published literature, setting of which are mainly based on theory and fitting based on the number of confirmed cases reported.^11,35–37^ The information in early outbreak is limited and circumstances can evolve rapidly. However, estimating risk must be produced within a short time period. To accurately simulate the epidemic, we estimated the parameters based on published literature and the number of confirmed cases reported in the initial stage of the epidemic.

Wuhan is the location of a central hub in China’s rail and aviation networks. The results of previous studies showed that the total risk coefficients of each province had a high correlation with the reported cumulative numbers of confirmed cases, which had effectiveness of risk evaluation on the initial situation of the outbreak.^18–20^ Hu et al. estimated the output risk of Wuhan based on the number of reported cases.^18^ It would be lower due to many COVID-19 cases being undocumented in January 2020.^38^ The previous studies assessed the risk of Wuhan output without considering the infectivity of asymptomatic persons.^19,20^ The output risk factors may be underestimated by approximately 15.5-20.2% without asymptomatic infections.^25^ Jia et al. developed a risk model that leveraged population flow from Wuhan, gross domestic product, and other variables to identify high risk regions.^39^ However, this study treated the risk of daily population migration as homogeneous. Our study assumed the risks of the same number of population moving out were obviously increasing, with the increase of the number of infections in Wuhan.^18^ In this study, the risk of asymptomatic infections was lower than other published estimates.^40^ Note that our value depends on the asymptomatic infections of epidemiological investigation instead of model simulation.

Earlier studies suggested that population movement would increase the risk of epidemic transmission. The export risks of COVID-19 varied from Wuhan to other provinces of China.^19^ Indeed, our model identified some provinces with a higher-than-expected level of infection, which suggested more community transmission, which was under-performing compared with the benchmark derived from the outflow population from Wuhan. On the other hand, the over-performing provinces with lower trends than expected might have implemented highly successful public health measures. Nevertheless, they may be prone to inaccurate data reporting.

This study showed that the total risk coefficients of the epidemic lagging by 5 d in each province that had the highest correlation with the reported cumulative numbers of cases reported. The result is close to the research of Hu et al.^41^ The lag time may be related to incubation period, and the time from onset to diagnosis. The assessment of the output risk provides a scientific reference for prediction and warning of the epidemic in each province. And the lag time is conducive to the timely management of epidemic prevention protocols and medical workers, planning of epidemic prevention materials, and implementing the containment strategy.

The results showed that, whether the implementation of travel restrictions of Wuhan city was delayed for 3 d, 5 d, and 7 d, the output risk would increase by approximately 40%, 55%, and 65%, respectively. In particular, the daily risk coefficients were the highest from the January 23 to the 28. The cause may be that this period is approaching the Spring Festival, and the travel intensity was higher. A series of public health measures, including travel restrictions of Wuhan city effectively, reduced the spread of the epidemic in a timely manner and curbed the development of the new spike in infections.^42^ On the basis of no new cases in Wuhan for a week, Wuhan lifted the traffic control measures on April 8, 2020.^43^

We focused on the relative strength of the epidemic risk in each province rather than the absolute number of cases, although we simulated the spread of COVID-19 in Wuhan by using reported data to calibrate the parameters. A contribution of our approach is to robustly characterize the relative risk and geographical distribution of COVID-19 over time. Moreover, our approach is generalizable to other datasets of population emigration. This method can facilitate policy decisions, such as the dynamic monitoring and allocation of resources and manpower.

## Limitations

This study has some limitations. First, the modified SEIR model is based on the assumption that Wuhan is relatively closed. So, it may impact on the accuracy of epidemic simulation. Second, the estimation of parameters of modified SEIR model is based on the official daily reported cumulative number of COVID-19 cases, which is relatively limited, such as when the pandemic started and the number of people infected at that time. The assessment should be updated in light of new evidence over time. Third, this study only considers the output risk from Wuhan city and does not consider the migration between other provinces and even countries. Fourth, our results are based on the assumption that the number of infected people is uniformly distributed in the out-migration population from Wuhan. Fifth, we do not consider other prevention and control measures other than travel restrictions at the beginning of the epidemic in Wuhan except the travel restrictions of Wuhan city, which may overestimate the output risk coefficients. Finally, this study was conducted on a provincial spatial scale and cannot be applied to smaller spatial scales.

## Conclusions

The present study estimated the output risk quantitatively and evaluated the travel restrictions of Wuhan city based on the modified SEIR infectious disease dynamic model. The export risks of COVID-19 varied from Wuhan to other provinces of China. This study demonstrated that the travel restrictions of Wuhan city had reduced the export risk and controlled the spread of the COVID-19 epidemic in China effectively in the early stages of the COVID-19 pandemic. The implementation of the measure may provide a reference for the prevention and control of the COVID-19 epidemic all over the world.
